# Role of adiponectin in diabetes myocardial ischemia-reperfusion injury and ischemic postconditioning ^1^

**DOI:** 10.1590/s0102-865020200010000007

**Published:** 2020-03-23

**Authors:** Chen Cao, Hui-min Liu, Wei Li, Yang Wu, Yan Leng, Rui Xue, Rong Chen, Ling-hua Tang, Qian Sun, Zhongyuan Xia, Qi-zhu Tang, Di-fei Shen, Qing-tao Meng

**Affiliations:** I PhD, Department of Endocrinology, 3rdHospital of Wuhan City, Wuhan, Hubei 430074, China. Conception and design of the study, final approval.; II PhD, Department of Anesthesiology, Renmin Hospital of Wuhan University, Wuhan, Hubei 430060, China. Conception and design of the study, final approval.; III PhD, Department of Anesthesiology, Renmin Hospital of Wuhan University, Wuhan, Hubei 430060, China. Technical procedures, final approval.; IV PhD, Department of Anesthesiology, Renmin Hospital of Wuhan University, Wuhan, Hubei 430060, China. Technical procedures, manuscript writing, critical revision, final approval.; V PhD, Department of Anesthesiology, Renmin Hospital, Hubei University of Medicine, Shiyan, Hubei 442000, China. Statistical analysis, final approval.; VI Master degree, Department of Anesthesiology, Renmin Hospital of Wuhan University, Wuhan, Hubei 430060, China. Reagents, materials, and analysis tools; final approval.; VII PhD, Department of Anesthesiology, Renmin Hospital of Wuhan University, Wuhan, Hubei 430060, China. Reagents, materials, and analysis tools; final approval.; VIII PhD, Department of Cardiovascularology, Renmin Hospital of Wuhan University, Wuhan, Hubei 430060, China. Final approval.; IX PhD, Department of Cardiovascularology, Renmin Hospital of Wuhan University, Wuhan, Hubei 430060, China. Scientific and intellectual content of the study, statistical analysis, final approval.; X PhD, Department of Anesthesiology, Renmin Hospital of Wuhan University, Wuhan, Hubei 430060, China. Scientific and intellectual content of the study, statistical analysis, final approval.

**Keywords:** Adiponectin, Diabetes, Reperfusion Injury, Ischemic Postconditioning

## Abstract

**Purpose:**

Patients with diabetes are vulnerable to myocardial I/R (ischaemia/reperfusion) injury, but are not responsive to IPO (ischaemic post-conditioning). We hypothesized that decreased cardiac Adiponectin (APN) is responsible for the loss of diabetic heart sensitivity to IPO cardioprotecton.

**Methods:**

Diabetic rats were subjected to I/R injury (30 min of LAD occlusion followed by 120 min of reperfusion). Myocardial infarct area was determined by TTC staining. Cardiac function was monitored by a microcatheter. ANP, 15-F2t-isoprostane, nitrotyrosine and MDA were measured by assay kits. Levels of p-Akt, total-Akt and GAPDH were determined by Western Blot.

**Results:**

Diabetic rats subjected to myocardial IR exhibited severe myocardial infarction and oxidative stress injury, lower APN in the plasma and cardiac p-Akt expression ( *P* <0.05). IPO significantly attenuated myocardial injury and up-regulated plasma APN content and cardiac p-Akt expression in non-diabetic rats but not in diabetic rats. Linear correlation analysis showed that the expression of adiponectin was positively correlated with p-Akt and negatively correlated with myocardial infarction area ( *P* <0.01).

**Conclusion:**

Protective effect of IPO was tightly correlated with the expression of adiponectin, exacerbation of I/R injury and ineffectiveness of IPO was partially due to the decline of adiponectin and inactivation of Akt in diabetes mellitus.

## Introduction

Over the last decades, diabetes mellitus (DM) has aroused increasing attention globally^1^ . Ischemic heart disease is a major cardiovascular complication of diabetes and the primary cause of mortality in diabetic disease^2^ . A number of studies have confirmed that compared with non-diabetic patients, patients with diabetes were susceptible to myocardial ischemia-reperfusion injury, and have poor treatment effect^3^ . The risk of myocardial infarction death is increased in diabetic patients compared to those without diabetes^4 , 5^ . Ischemic postconditioning provides protective effects against ischemia/reperfusion injuries and this has been confirmed by lots of studies, but postconditioning seems to lose its cardioprotective effect in subjects with diabetes, while the underlying mechanism is largely unknown.

As a cytokine secreted by fat cells, adiponectin (APN) plays an important role in regulating metabolism, blood vessels, and cardiac protection mechanism^6^ . Studies have shown that myocardial ischemia-reperfusion injury was significantly increased in adiponectin gene knockout mice^7^ , and adiponectin level was significantly decreased in patients with DM, and they had a negative correlation with ischemic heart disease^8^ . All these studies suggested that low adiponectin level might be one of the important mechanisms of the occurrence and development in diabetic patients with ischemic heart disease. However, whether ANP expression was inhibited in diabetic heart remains unclear. Ischemic postconditioning (IPO) has been demonstrated to be beneficial following ischemia/reperfusion (IR) injury in the heart, brain, liver, kidney, and spinal cord, and its mechanical interventions against reperfusion injury relate to multiple and interacting components in endogenous protective mechanisms. In the present study, we tested the relations of ANP expression to ischemia reperfusion injury, and investigated the potential mechanisms of ineffectiveness of ischemic postconditioning on diabetic myocardium ischemia reperfusion injury.

## Methods

### Induction of diabetes and myocardial ischemic model

All study protocols were in accordance with internationally accepted principles and the Guidelines for the Care and Use of Laboratory Animals of Wuhan University, Wuhan, People’s Republic of China. The study was reviewed and approved by the Research Ethics Committee of Renmin Hospital of Wuhan University.

This study compiled with the Guide for the Care and Use of Laboratory Animals by the National Institutes of Health (NIH Publication No. 80-23) and was approved by Bioethics Committee of Renmin Hospital of Wuhan University (Wuhan, China). Adult male Sprague-Dawley rats (250 ± 10g, aged 6-8 weeks of age) were obtained from HUNAN SLAC JD Laboratory Animal Co. Ltd. All the animals were randomly divided into 6 groups: normal sham group (group NS, n=8), normal ischemia reperfusion group (group NIR, n=8), normal ischemia postconditioning group (group NIPO, n=8), diabetic mellitus sham group (group DMS, n=8), diabetic mellitus ischemia reperfusion group (group DMIR, n=8) and diabetic mellitus ischemic postconditioning group (group DMIPO, n=8). After 3 days of acclimatization, the rats were fasted 12 h for type I diabetes induction. The diabetes model was induced via a single intraperitoneal injection of 60 mg/kg Streptozotocin (STZ), as described previously. Only those rats showing hyperglycaemia (blood glucose level ≥16.7 mmol/l at least three times) were considered Type I diabetic.

Myocardial ischemia reperfusion (I/R) injury model was established by the LAD coronary artery occlusion for 30 min followed by reperfusion for 2 h. Sham-operated rats (NS group and DMS group) underwent the same surgical procedures, but without ligation. Ischaemia (NIR group and DMIR group) was verified by discolouration of the ischaemic zone and elevation of ST-segment with limb lead II. IPO (NIPO group and DM + IPO group) was induced by 3 cycles of reperfusion (10 s) and re-occlusion (10 s) before 120 min of reperfusion. At the end of reperfusion, blood and tissue samples were collected for further analysis.

### Determination of myocardial infarct area

The infarction area was measured by Evans blue and 2,3,5-Triphenyltetrazolium chloride (TTC) double staining: after the animals were sacrificed, the ligature around the coronary artery was reoccluded and 2mL of 0.25% Evens Blue dye were injected into the aorta to map the normally perfused region of the heart. The myocardial area at risk (AAR) for infarction was delineated by the area of myocardium not dyed. The presence of Evans blue was used to identify the area that was not subjected to the ischemia. Rat hearts were rapidly excised and frozen at −20^°^C and then sliced into 2mm thick sections perpendicular to the heart base-apex axis using a heart slice chamber. The slices were incubated for 15min at 37^°^C in a phosphate-buffered 1% TTC (Sigma-Aldrich, St. Louis, MO) solution to determine infarcted myocardium. The viable tissue was stained red by TTC, while the infarct portion not taking up TTC stain remained pale. The infarct size was determined by an image analysis system (Image-Pro Plus 3.0, Media Cybernetics, MA). Two researchers scored the slides independently to ensure reliability of the results. The percentage of area at risk versus left ventricle (AAR/LV ×100%) and infarct area versus area at risk (IA/AAR × 100%) were calculated.

### Assessment of cardiac function

Myocardial function was intermittently monitored at the baseline and after I/R or IPO procedure. A microcatheter was inserted into the left ventricle to measure the cardiac function. The artery pressure was measured by right common carotid artery intubation. The heart rate (HR), left ventricular systolic pressure (LVSP) and the instantaneous first derivation of LVP (+dp/dt maximum and -dp/dt maximum) were monitored by electrophysiolograph (BioPAC, MH150, USA). All data were derived by AcqKnowledge 4.0 software.

### Measurement of adiponectin in the plasma

Blood samples were collected at the end of reperfusion and centrifuged at 3,000 g, for 10 min at 4˚C. Serum was separated and stored at –20˚C. ANP levels were measured using an ANP assay kit (Boster Biological Technology, Wuhan, China) according to the manufacturer’s instructions.

### Western Blot analysis

Myocardium tissue samples (100mg) were homogenized in lysis buffer with electric homogenate machine, and then the homogenates were centrifuged at 12,000rpm for 15mins at 4°C. After determining with BCA protein assay kit (Beyotime Biotech Inc., Jiangsu, China), the supernatants were used as protein samples. Samples containing equal amounts were separated on a 10% SDS-polyacrylamide gel, and then proteins were transferred to PVDF membrane. After blocked in 5% nonfat-dried milk for 1h, membranes were incubated with rabbit anti-phospho-Akt (ser473) and anti-total-Akt monoclonal antibody (1:1000 dilution, Cell Signaling Technology, USA) overnight at 4°C. Subsequently, the membranes were washed and incubated with the corresponding fluorescent tags goat anti-rabbit polyclonal IgG (1:10000 dilution, LI-COR, USA) for 1h at room temperature. Then, the Odyssey scanner was open and the membranes were put facing down on the designated area for infrared fluorescence detection on the Odyssey Imaging Systems (LI-COR, USA). GADPH was chosen as a loading control to further assure the same volume for all the samples.

### Measurement of 15-F2t-isoprostane

Serum and cardiac 15-F2t-isoprostane were special indexes of oxidative stress induced by lipid peroxidation in heart. Serum was collected from arterial blood at the end of reperfusion. After reperfusion, hearts were removed rapidly and frozen in liquid nitrogen. Ventricle tissue was sampled and homogenized in lysis buffer. The samples were homogenized and centrifuged at 12 x 10^3^g for 15 minutes at 4°C. Supernatants were collected. Serum and cardiac 15-F2t-isoprostane were then measured by using an ELISA kit (Cayman Chemical, USA) according to manufacturer’s instructions.

### Measurement of myocardial nitrotyrosine and MDA

Total protein concentration in the lysis was determined by protein assay kit. The levels of myocardial nitrotyrosine in the collected supernatant were assayed by the nitrotyrosine assay kit according to the manufacturer’s instructions (Millipore, USA). Malondialdehyde (MDA) was analyzed spectrophotometrically according to the instructions of the assay kits (Nanjing Jiancheng Bioengineering Institute, China).

### Statistical analysis

The data are expressed as mean±s.d. The GraphPad Prism 5.0 statistical software program (GraphPad Software, San Diego, CA, USA) was used to set up our database and to calculate the results. Statistical evaluation of the data was performed by one way analysis of variance (ANOVA), followed by Tukey’s *post hoc test* . *P* <0.05 was considered as statistically significant.

## Results

### General characteristics of the experimental animals before MI/R injury

As shown in Table 1 , after 8 weeks of STZ injection, type 1 diabetic rats exhibited characteristic symptoms of diabetes, including hyperglycemia, polydipsia, polyphagia, weight loss, increased blood glucose levels compared with age-matched non-diabetic rats.

**Table 1:** General characteristics of each group.

Parameters /Group	N	D
Blood glucose(mM)	5.86±1.15	27.45±4.06^*^
Body weight (g)	482.7±36.37	248.6±28.51^*^

N: Non-diabetic rats; D: STZ-induced diabetic rats. Results are expressed as mean ± SD. * *p* < 0.05 *vs* . N group, n= 8.

### The protective effect of IPO on myocardial ischemia reperfusion injury was blocked in DM myocardial

**Figure 1:**
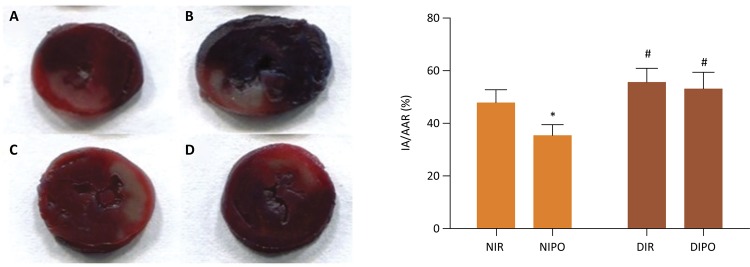
The effect of IPO on myocardial infarcted area on N and DM rats subjected to IR in the presence and absence of IPO. The blue-stained areas represent non-ischemic tissue, red-stained areas represent the area at risk (AAR), and pale areas represent infarct areas (IA). Values are expressed as mean ± SD (n=8). * *P* < 0.05, # *P* < 0.05 *vs* . NIR group.

**Figure 2:**
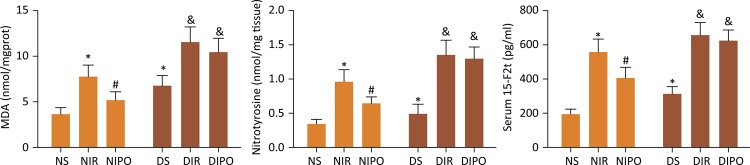
- The level of MDA and Nitrotyrosine in myocardium and serum level of 15-F2t-isoprostane in N and DM rats subjected to IR in the presence and absence of IPO. Values are expressed as mean ± SD (n=8). * *P* < 0.05 *vs* . NS group, # *P* < 0.05 *vs* . NIR group.

**Figure 3:**
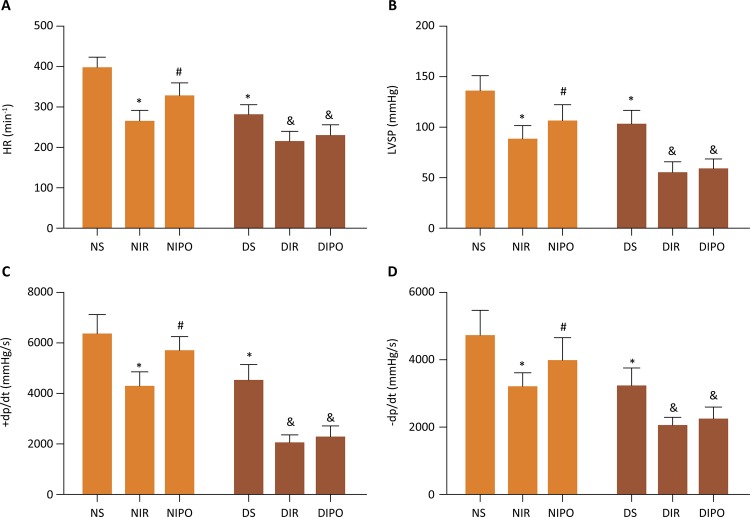
- The change of cardiac function on N and DM rats subjected to IR in the presence and absence of IPO. Values are expressed as mean ± SD (n=8). * *P* < 0.05 *vs* . NS group, # *P* < 0.05, & *P* < 0.05 *vs* . NIR group.

In order to investigate the underlying protective mechanisms of IPO, we studied the myocardial infarcted area subjected to IR in the presence and absence of IPO on N and DM rats. The infarct size in NIPO rats remarkably decreased in comparison with that in NIR rats ( *P* <0.05), the infarct size in DMIR and DMIPO rats remarkably increased in comparison with that in NIR rats ( *P* <0.05). Moreover, there was no difference between DMIR and DMIPO rats ( *P* >0.05) ( Fig. 1 ). To identify the possible mechanisms underlying the cardioprotective effects of IPO, we evaluated serum and cardiac 15-F2t-isoprostane as cardiac special index of oxidative stress. We further measured MDA and nitrotyrosine levels as indicators of oxidative stress. As shown in Figure 2 , IR induced oxidative stress in both normal and diabetic rats as demonstrated by significantly increased levels of MDA, nitrotyrosine and 15-F2t-isoprostane in both serum and cardiac. IPO attenuated the IR induced oxidative stress in normal ( *P* <0.05) but not diabetic rats ( *P* >0.05). Next, we examined the change of cardiac function in the presence and absence of IPO. The levels of HR, LVSP, +dp/dt and -dp/dt were remarkably decreased in the NIR, NIPO and DMS rats in comparison with those in NS group ( *P* <0.05). The levels of HR, LVSP, +dp/dt and -dp/dt in NIPO were increased in comparison with those in NIR group ( *P* <0.05). But there was no difference for the levels of HR, LVSP, +dp/dt and -dp/dt between DMIR and DMIPO groups ( *P* >0.05) ( Fig. 3 ).

### Adiponectin related p-Akt and total-Akt expressions is crucial in the protection of IPO

**Figure 4:**
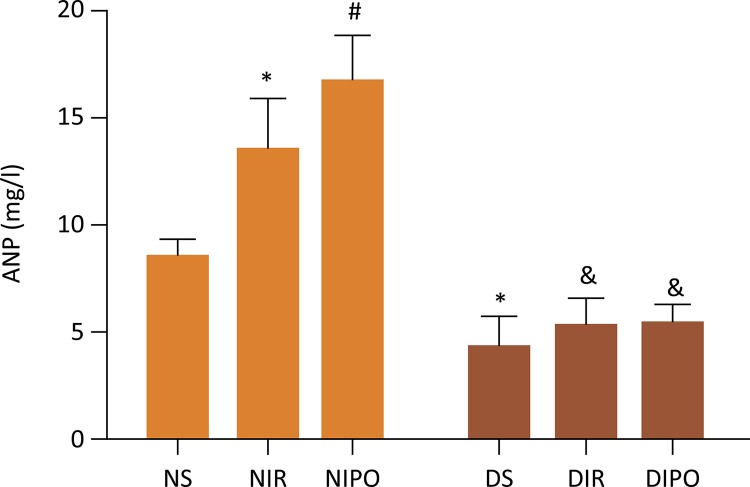
The level of plasma adiponectin in N and DM rats subjected to IR in the presence and absence of IPO. Values are expressed as mean ± SD (n=8). * *P* < 0.05 *vs* . NS group, # *P* < 0.05 *vs* . NIR group.

**Figure 5:**
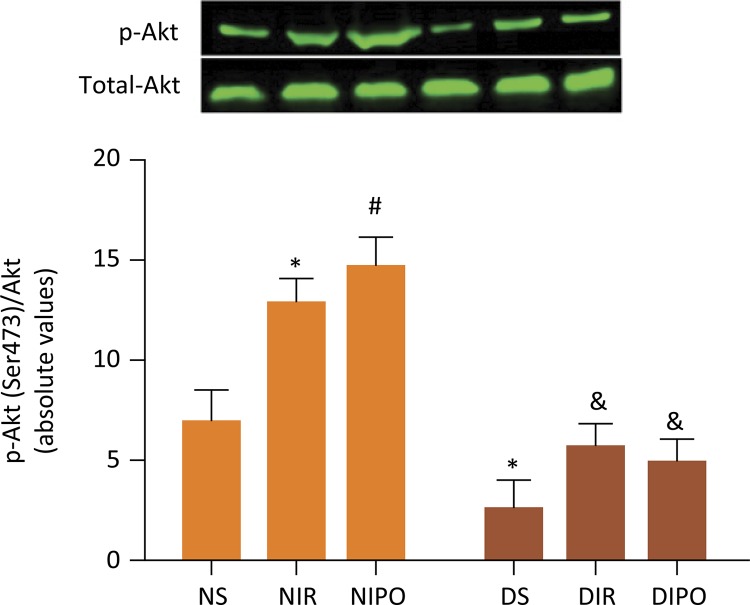
- The change of p-Akt and total-Akt expression in the presence and absence of IPO. Values are expressed as mean ± SD (n=8). * *P* < 0.05 *vs* . NS group, # *P* < 0.05 *vs* . NIR group.

To determine the effect of adiponectin on the protection of IPO, we examined the content of adiponectin in the plasma and we observed that the NIR and NIPO induced a remarkable increase for ANP level when compared with that in NS group ( *P* < 0.05), associated with a decrease in ANP in DMS group ( *P* <0.05). However, there was no difference among DMS, DMIR and DMIPO rats ( *P* >0.05) ( Fig. 4 ). Next, we investigated the changes of p-Akt and total-Akt expression in the presence and absence of IPO. The results showed that the NIR and NIPO induced a remarkable increase in p-Akt In comparison with that in NS group ( *P* < 0.05), associated with a decrease in p-Akt in the DMS group ( *P* <0.05). There was no difference for the expressions of p-Akt in DMS, DMIR and DMIPO groups ( *P* >0.05), and there was no difference for the expressions of t-Akt in all groups ( *P* >0.05) ( Fig. 5 ).

**Figure 6:**
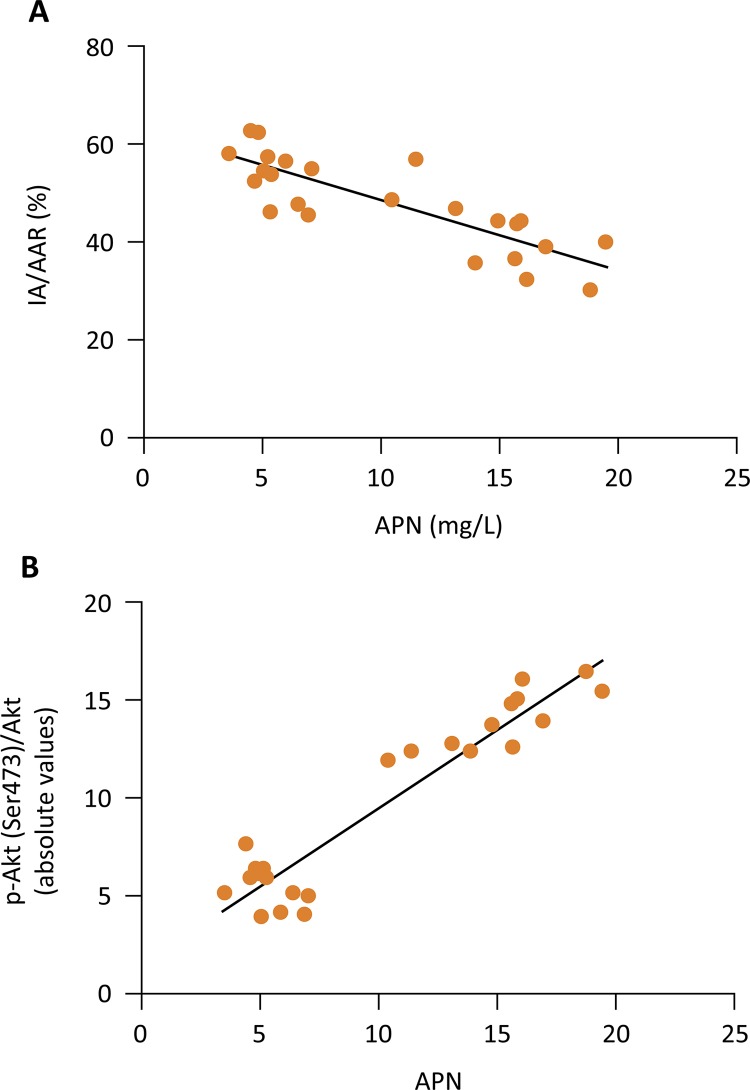
The relationship between ANP and myocardial infarcted area, P-Akt. The simple linear regression between plasma adiponectin levels and myocardial infarct size together with p-Akt/ Akt protein expression in N and DM rats subjected to IR, with or without IR.

Moreover, we analyzed the relationship between ANP and myocardial infarcted area: p-Akt with simple linear regression ( Fig. 6 ). The expression of ANP was positively correlated with p-Akt with R^2^ at 0.89 ( *P* < 0.001), while myocardial infarct size was negatively correlated with ANP: the corresponding R^2^ was 0.69 ( *P* <0.001).

## Discussion

We have demonstrated that IPO significantly decreased the myocardial infarction area and oxidative stress induced by MIR injury in the normal rats, which was intensely associated with an enhanced expression of ANP and p-Akt. The beneficial effects of IPO were abolished in diabetic rats, while the change of ANP and p-Akt was also removed in diabetic rats. Our results also showed that although ANP expression was upregulated under myocardial ischemia and reperfusion injury in normal rats, it was not enough to resist myocardial insult resulted from ischemia reperfusion in diabetic rats, while postconditioning could not increase the expression of APN and p-Akt after MIR injury. Our results are well in line with the findings of Christopher^9^ . These findings suggest that ischemia-reperfusion injury and protective effect of IPO was tightly correlated with the expression of ANP, exacerbation of ischemia-reperfusion injury, and ineffectiveness of IPO was partially due to the decline of ANP and inactivation of PI3K/Akt signal pathway in diabetes mellitus.

APN was a new kind of cDNA cloned by Scherer^6^ from the fat cells of rats, capable of three biological functions estimated by experiments and clinical researches^7^ in the past decades: insulin sensitizing or metabolic regulations (mainly of the liver and muscle), anti-inflammatory or blood vessel protection, and anti-ischemia myocardial protection. High expressions of APN, by the anti-inflammatory mediatory role of macrophages, were able to stop the exacerbation of metabolic and cardiovascular diseases^8^ . Surveys of epidemics showed that, among the patients suffering from obesity and diabetes accompanied by hyperglycemia, the lower the APN level the higher the risk of cardiovascular diseases. There was a negative correlation between the plasma APN density and insulin level. It was demonstrated that average adiponectin levels decreased but excessive oxidative stress increased in diabetes. The multiple mechanisms involved, such as NADPH oxidase, xanthine oxidase and mitochondrial respiratory chain can be inhibited by interventions such as exercise and medication. The expressions of APN were regulated by some inflammatory factors, transcription factors and hormones, e.g., the inflammatory factors of TNF-alpha. IL-6 restrained the expression and secretion of APN, among which the TNF-alpha functioned by intensely restraining the activity of APN promoters. At the state of obesity, most fat factors were up-regulated, acting as a pre-inflammatory mediator, promoting the process of the disease. On the contrary, APN was down-regulated because of obesity^10^ . Walch^11 , 12^ proved that APN knockout mice were more susceptible to myocardial ischemia reperfusion injury, and that with APN recombinant before the reperfusion, the myocardial infarction area could be prominently reduced. These results have shown that among the type II diabetic patients, the lower level of APN not only resulted in ischemia myocardial diseases, but also accelerated the death of myocardial cells after ischemia reperfusion. Results of this study have also shown that ischemia reperfusion, especially ischemic postconditioning, would result in APN ascending, greatly reducing the myocardial infarction area. However, in the diabetic myocardium, the expressions of APN were restrained and could not up-regulate both the ischemia reperfusion injury and post processing, leading to a larger area of myocardial infarction. It was also indicated that the myocardial infarction area could not be decreased in diabetic patients accompanied by acute myocardial infarction by ischemic postconditioning, which would enlarge the infarct area and deteriorate the prognosis. Our results also confirmed that the protective effect of IPO on myocardial ischemia reperfusion injury was blocked in DM myocardial,

The PI3K/Akt signal pathway was an important factor for the maintenance of cellular cycling, restraining cell death, and promoting cell growth and proliferation. In myocardial cells, the activation of the PI3K/Akt pathway could reduce the calcium overload, maintain the stability of mitochondria membranes, and prevent subsequent oxidation damage and apoptosis resulting from the mitochondria mPTP opening, serving as an internal anti-oxidation defense of myocardial cells^13^ . In addition, ischemic postconditioning, an important internal protector, could activate the PI3K/Akt pathway. The studies have shown that the protections of ischemic postconditioning lost efficacy after the diabetic myocardial ischemia reperfusion, whose mechanism was proved to be intensely correlated with the inactivation of the PI3K/Akt pathway^14 , 15^ . Tingting^16^ proved that N-acceltycystine (NAC) and allopurinol (ALLO) synergistically reduced the myocardial ischemia reperfusion injury of diabetic rats by up-regulating the PI3K/Akt pathway. This study has shown that ANP expression would be upregulated under myocardial ischemia and reperfusion injury in normal rats, but it was not enough to resist myocardial insult resulted from ischemia reperfusion in diabetic rats, while postconditioning could not increase the expression of APN and p-Akt after MIR injury. We also confirmed that the expression of ANP was positively correlated with p-Akt, while myocardial infarct size was negatively correlated with ANP expressions.

The present study showed that APN levels negatively correlated to myocardial injury and positively correlated to p-Akt levels. Further studies are needed to determine the relationship between APN and Akt activation in vitro. Our experimental findings are only instructive for myocardial protection. It is reported that eNOS and STAT3 play a role in myocardial I/R injury in diabetic rats. Further studies are necessary to determine the relationship between APN and activation of eNOS and STAT3 in vitro.

## Conclusion

The ischemia-reperfusion injury and protective effect of IPO was tightly correlated with the expression of ANP and exacerbation of ischemia-reperfusion injury. In addition, ineffectiveness of IPO was partially due to the decline of ANP and inactivation of the PI3K/Akt signal pathway in diabetes mellitus.

## Footnotes

1 Research performed at Central Laboratory, and Department of Anesthesiology, Renmin Hospital, Wuhan University, Wuhan, Hubei, China.
